# Supplementary material for: Translation, cross-cultural adaptation and validation of Patient Satisfaction with Pharmacist Services Questionnaire (PSPSQ 2.0) into the Nepalese version in a community settings
Source: PLoS One. 2020 Oct 9;15(10):e0240488. doi: 10.1371/journal.pone.0240488 (PMC7546480; doi:10.1371/journal.pone.0240488)
Supplement: S1 File — (DOC) [file pone.0240488.s001.doc]

Pt. #

**PSPSQ 2.0**

***Please complete this survey by checking the option that best describes your opinion:***

|  | **Quality of Care** | Strongly Agree | Agree | Disagree | Strongly Disagree |
| --- | --- | --- | --- | --- | --- |
| 1 | The pharmacist fully addressed the main health reason/concerns/issues during my visit. | 4 | 3 | 2 | 1 |
| 2 | The pharmacist was professional in all of our interactions. | 4 | 3 | 2 | 1 |
| 3 | The pharmacist explained information to me in a manner that I could understand. | 4 | 3 | 2 | 1 |
| 4 | The pharmacist checked to see if I understood all the information. | 4 | 3 | 2 | 1 |
| 5 | The pharmacist spent as much time necessary to help me with my questions and concerns. | 4 | 3 | 2 | 1 |
| 6 | The pharmacist made sure I understood how important it is to follow the drug regimen. | 4 | 3 | 2 | 1 |
| 7 | The pharmacist provided useful recommendations on how to take my medications. | 4 | 3 | 2 | 1 |
| 8 | The pharmacist provided useful recommendations about managing my overall health (e.g. diet, exercise). | 4 | 3 | 2 | 1 |
| 9 | The pharmacist worked with me to manage my medication related issues (e.g. cost, side effects of drugs). | 4 | 3 | 2 | 1 |
| 10 | The pharmacist followed up on my progress in a timely manner. | 4 | 3 | 2 | 1 |
|  | | | | | |
|  | **Interpersonal Relationship (pharmacist/patient)** | Strongly Agree | Agree | Disagree | Strongly Disagree |
| 11 | The pharmacist was caring and kind in dealing with my health issues. | 4 | 3 | 2 | 1 |
| 12 | The pharmacist encouraged me to achieve my treatment goals. | 4 | 3 | 2 | 1 |
| 13 | I felt comfortable in my interactions with the pharmacist. | 4 | 3 | 2 | 1 |
| 14 | The pharmacist was respectful to me during our interactions. | 4 | 3 | 2 | 1 |
| 15 | The pharmacist was committed to improving my health. | 4 | 3 | 2 | 1 |
| 16 | I could trust the information that the pharmacist provided. | 4 | 3 | 2 | 1 |
|  | | | | | |
|  | **Overall** | Strongly Agree | Agree | Disagree | Strongly Disagree |
| 17 | I was satisfied with the overall care provided by my pharmacist. | 4 | 3 | 2 | 1 |
| 18 | I would recommend my pharmacist to people I know. | 4 | 3 | 2 | 1 |
| 19 | If needed, I would continue seeing this pharmacist for my healthcare needs. | 4 | 3 | 2 | 1 |

| 20 | The overall care provided by the pharmacist. | Exceeded my expectations  4 | Met my expectations  3 | Did not meet my expectations  2 | Had no expectations  1 |
| --- | --- | --- | --- | --- | --- |

Copyrighted Law and Bounthavong 2007 revised 2014
