# Supplementary material for: Translation, cross-cultural adaptation and validation of Patient Satisfaction with Pharmacist Services Questionnaire (PSPSQ 2.0) into the Nepalese version in a community settings
Source: PLoS One. 2020 Oct 9;15(10):e0240488. doi: 10.1371/journal.pone.0240488 (PMC7546480; doi:10.1371/journal.pone.0240488)
Supplement: S2 File — (PDF) [file pone.0240488.s002.pdf]

क्रम संख्या. #

फार्मासिस्टको सेवा सँग बिरामीको सन्तुष्टि प्रश्नावली २.०

कृपया यो सर्वेक्षणको लागि तपाईलाई सबैभन्दा मिल्ने खालको विकल्पहरु छानी पुरा गर्नुहोस् ।

	सेवाको गुणस्तर	धेरै सहमत छु ।	सहमत छु ।	असहमत छु ।	धेरै असहमत छु ।
१	फर्मासिस्टले मेरो भ्रमणको समयमा मुख्य स्वास्थ्य कारण/चिन्ता/समस्यालाई पूर्णरुपमा सम्बोधन गर्नुभयो |	४	३	२	१
२	हामीबिचको सम्पूर्ण अन्तरक्रियामा फर्मासिस्ट आफ्नो पेशामा व्यावसायिक पाइयो ।	४	३	२	१
३	फर्मासिस्टले मैले बुझ्ने तरिकाले सुचना प्रदान गर्नुभयो ।	४	३	२	१
४	मैले सबै जानकारी बुझे कि नाइँ भनेर फर्मासिस्टले पुनं निरीक्षण गर्नुभयो |	४	३	२	१
५	मेरो प्रश्न र जिज्ञासासँग मलाई मदत गर्न फार्मासिस्टले आवश्यक समय बिताउनुभयो |	४	३	२	१
६	फर्मासिस्टले औषधी प्रयोग गर्ने बिधी कति महत्वपूर्ण हुन्छ भन्ने मलाई बुझाउन निश्चित गर्नुभयो |	४	३	२	१
७	फर्मासिस्टले मलाई औषधी लिने प्रक्रियाको बारेमा उपयोगी सुझावहरू दिनुभयो ।	४	३	२	१
८	समग्र रूपमा शरीरलाई स्वस्थ बनाइराख्न चाहिने कुरा (जस्तैः खानपिन, शारीरिक ब्यायाम) बारेमा पनि फर्मासिस्टले मलाई उपयोगी सुझावहरू दिनुभयो ।	४	३	२	१
९	फर्मासिस्टले मेरो औषधि सम्बन्धी समस्याहरु (जस्तैः लागत, औषधिका नकारात्मक असरहरु) व्यवस्थापन सँगै मिलेर काम गर्नुभयो |	४	३	२	१
१०	फर्मासिस्टले उपचार पश्चात् मेरो स्वास्थ्यमा देखिएका प्रगति बारेमा समय-समयमा जानकारी लिइरहनु भयो ।	४	३	२	१

	पारस्परिक सम्बन्ध (फर्मासिष्ट / बिरामी)	धेरै सहमत छु ।	सहमत छु ।	असहमत छु ।	धेरै असहमत छु ।
११	मेरो स्वास्थ्य समस्याहरु समाधान गर्न फर्मासिस्ट हेरचाह र दयालु थिए |	४	३	२	१
१२	मेरो उपचार लक्ष्य प्राप्त गर्नको लागि फार्मासिस्टले प्रोत्साहन दिनुभयो |	४	३	२	१
१३	मैले फार्मासिस्टसँगको अन्तर्क्रियामा सहज महसुस गरे |	४	३	२	१
१४	हाम्रो अन्तरक्रियामा मप्रति फर्मासिस्ट सम्मानजनक रहेको पाएँ |	४	३	२	१
१५	मेरो स्वास्थ्य सुधारको लागि फर्मासिस्ट सधैं प्रतिबद्ध हुनुभयो ।	४	३	२	१
१६	फर्मासिष्टद्वारा प्रदान गरिएका जानकारीहरुमा म विश्वास गर्न सक्थें ।	४	३	२	१

	समग्रमा	धेरै सहमत छु ।	सहमत छु ।	असहमत छु ।	धेरै असहमत छु ।
१७	फर्मासिस्टद्वारा प्रदान गरिएका सम्पूर्ण सेवामा म सन्तुष्ट थिएँ |	४	३	२	१
१८	मैले चिनेका ब्यक्तिहरुलाई म मेरो फर्मासिस्टलाई सिफारिस गर्नेछु |	४	३	२	१
१९	आवश्यक परेमा, स्वास्थ्य सेवासम्बन्धी आवश्यकता पूरा गर्न म फर्मासिस्टलाई भेटिरहने छु |	४	३	२	१

२०	समग्रमा फर्मासिस्टद्वारा प्रदान गरिएका सेवाहरु	मेरो अपेक्षाहरुभन्दा अधिक भयो । ४	मेरो अपेक्षाहरु पूरा भए । ३	मेरो अपेक्षाहरु पूरा भएनन् । २	अपेक्षाहरु थिएनन् । १

प्रतिलिपी अधिकार Law र Bounthavong २००७ संशोधित २०१४
