# Supplementary material for: Translation, cross-cultural adaptation and validation of Patient Satisfaction with Pharmacist Services Questionnaire (PSPSQ 2.0) into the Nepalese version in a community settings
Source: PLoS One. 2020 Oct 9;15(10):e0240488. doi: 10.1371/journal.pone.0240488 (PMC7546480; doi:10.1371/journal.pone.0240488)
Supplement: S1 Table — (DOCX) [file pone.0240488.s004.docx]

**S1 Table. Correlation Matrix**

|  | **Q1** | **Q2** | **Q3** | **Q4** | **Q5** | **Q16** | **Q17** | **Q18** | **Q19** | **Q20** |
| --- | --- | --- | --- | --- | --- | --- | --- | --- | --- | --- |
| **Correlation** | | | | | | | | | | |
| Q1 | 1.000 | .536 | .408 | .242 | .351 | .024 | -.072 | -.014 | -.033 | -.038 |
| Q2 | .536 | 1.000 | .377 | .285 | .378 | .042 | -.051 | .068 | .038 | .049 |
| Q3 | .408 | .377 | 1.000 | .632 | .573 | -.099 | -.083 | -.057 | -.042 | -.078 |
| Q4 | .242 | .285 | .632 | 1.000 | .745 | -.001 | -.013 | .050 | .012 | .010 |
| Q5 | .351 | .378 | .573 | .745 | 1.000 | .003 | .045 | .071 | .011 | .101 |
| Q6 | .299 | .225 | .319 | .534 | .735 | .084 | .097 | .033 | -.014 | .075 |
| Q7 | -.024 | .198 | .148 | .278 | .298 | -.094 | .039 | .124 | .088 | .066 |
| Q8 | .383 | .373 | .517 | .553 | .507 | .017 | .000 | .001 | -.056 | .017 |
| Q9 | -.039 | .032 | -.013 | .136 | .149 | -.013 | -.035 | -.027 | .003 | .036 |
| Q10 | .266 | .278 | .318 | .360 | .496 | .005 | -.020 | -.069 | -.028 | .030 |
| Q11 | .028 | .019 | .023 | -.087 | -.024 | .031 | .018 | -.109 | -.131 | .075 |
| Q12 | -.023 | -.085 | -.069 | -.078 | -.036 | .235 | .343 | .232 | .084 | .015 |
| Q13 | .103 | .087 | .112 | .079 | .110 | .235 | .043 | .217 | .117 | -.015 |
| Q14 | .111 | .075 | .048 | .096 | .098 | .273 | .112 | .120 | .241 | -.043 |
| Q15 | .022 | .044 | -.025 | .042 | .071 | .566 | .307 | .250 | .055 | .019 |
| Q16 | .024 | .042 | -.099 | -.001 | .003 | 1.000 | .358 | .296 | .193 | .076 |
| Q17 | -.072 | -.051 | -.083 | -.013 | .045 | .358 | 1.000 | .486 | .352 | .328 |
| Q18 | -.014 | .068 | -.057 | .050 | .071 | .296 | .486 | 1.000 | .528 | .138 |
| Q19 | -.033 | .038 | -.042 | .012 | .011 | .193 | .352 | .528 | 1.000 | .215 |
| Q20 | -.038 | .049 | -.078 | .010 | .101 | .076 | .328 | .138 | .215 | 1.000 |
| **Sig. (1-tailed)** | | | | | | | | | | |
| Q1 |  | .000 | .000 | .000 | .000 | .299 | .056 | .379 | .237 | .199 |
| Q2 | .000 |  | .000 | .000 | .000 | .178 | .129 | .066 | .203 | .140 |
| Q3 | .000 | .000 |  | .000 | .000 | .014 | .034 | .104 | .180 | .042 |
| Q4 | .000 | .000 | .000 |  | .000 | .495 | .390 | .137 | .397 | .416 |
| Q5 | .000 | .000 | .000 | .000 |  | .472 | .162 | .060 | .405 | .013 |
| Q6 | .000 | .000 | .000 | .000 | .000 | .033 | .016 | .235 | .378 | .050 |
| Q7 | .302 | .000 | .001 | .000 | .000 | .019 | .197 | .003 | .027 | .073 |
| Q8 | .000 | .000 | .000 | .000 | .000 | .357 | .498 | .495 | .109 | .355 |
| Q9 | .194 | .238 | .391 | .001 | .001 | .390 | .222 | .278 | .471 | .215 |
| Q10 | .000 | .000 | .000 | .000 | .000 | .459 | .334 | .065 | .271 | .256 |
| Q11 | .269 | .338 | .310 | .027 | .298 | .245 | .349 | .008 | .002 | .049 |
| Q12 | .305 | .030 | .064 | .042 | .213 | .000 | .000 | .000 | .032 | .370 |
| Q13 | .011 | .028 | .007 | .041 | .007 | .000 | .170 | .000 | .005 | .367 |
| Q14 | .007 | .049 | .148 | .017 | .016 | .000 | .007 | .004 | .000 | .175 |
| Q15 | .317 | .168 | .289 | .175 | .059 | .000 | .000 | .000 | .114 | .336 |
| Q16 | .299 | .178 | .014 | .495 | .472 |  | .000 | .000 | .000 | .047 |
| Q17 | .056 | .129 | .034 | .390 | .162 | .000 |  | .000 | .000 | .000 |
| Q18 | .379 | .066 | .104 | .137 | .060 | .000 | .000 |  | .000 | .001 |
| Q19 | .237 | .203 | .180 | .397 | .405 | .000 | .000 | .000 |  | .000 |
| Q20 | .199 | .140 | .042 | .416 | .013 | .047 | .000 | .001 | .000 |  |

*Determinant = 0.000*

*Each factor details from Q1 to Q2 is available in Supplementary file. S1 PSPSQ 2.0 Original version*
