# Supplementary material for: Translation, cross-cultural adaptation and validation of Patient Satisfaction with Pharmacist Services Questionnaire (PSPSQ 2.0) into the Nepalese version in a community settings
Source: PLoS One. 2020 Oct 9;15(10):e0240488. doi: 10.1371/journal.pone.0240488 (PMC7546480; doi:10.1371/journal.pone.0240488)
Supplement: Attachment — Submitted filename: Response to Reviewers.docx [file pone.0240488.s005.docx]

**August 13, 2020**

**Point to point response to the reviewers' comments**

We are very grateful for the reviews provided by the editor and the reviewers on our manuscript. The comments are encouraging, and the reviewers appear to share our judgment that this study and its results are interesting and relevant. Please kindly see below, in blue, our detailed response to comments.

All page numbers and line numbers refer to the manuscript file with tracked changes.

Sunil Shrestha and co-authors.

**Journal Requirements:**

When submitting your revision, we need you to address these additional requirements.

1. Please ensure that your manuscript meets PLOS ONE's style requirements, including those for file naming. The PLOS ONE style templates can be found at <https://journals.plos.org/plosone/s/file?id=wjVg/PLOSOne_formatting_sample_main_body.pdf> and <https://journals.plos.org/plosone/s/file?id=ba62/PLOSOne_formatting_sample_title_authors_affiliations.pdf>

**Authors' Response:** We have gone through both above-mentioned links and now kept manuscript as required by the PLOS ONE.

2. Please provide additional details regarding the consent of the expert reviewers employed to test the validation of this questionnaire. In the ethics statement in the Methods and online submission information, please ensure that you have specified (1) whether consent was informed and (2) what type you obtained (for instance, written or verbal, and if verbal, how it was documented and witnessed). If the need for consent was waived by the ethics committee, please include this information.

**Authors' Response:** The experts were given an Information Sheet and consent were obtained from them to be part of the expert committee. We have elaborated this thing in the Methods section (sub-section: Ethics statement) of the manuscript.

3. Please ensure that you have specified whether participant consent was informed. In addition, please refrain from stating p values as .000, either report the exact value or employ the format p<0.001.

**Authors' Response:** We have now reported the exact values of *p* in this manuscript.

4. We noticed you have some minor occurrence of overlapping text with the following previous publication(s), which needs to be addressed:

<https://fulltxt.org/article/957>
<https://mhealth.jmir.org/2018/1/e24/>

In your revision ensure you cite all your sources (including your own works), and quote or rephrase any duplicated text outside the methods section. Further consideration is dependent on these concerns being addressed.

**Authors' Response:** We have revised our manuscript by removing the overlapping text, rephrasing the duplicate text and giving the proper citation to the previously published work.

5. Thank you for stating the following in the Competing Interests section: "The author(s) declare that they have no competing interests." We note that one or more of the authors are employed by a commercial company: Jeevee Health Pvt Ltd.

**Authors' Response:** Santosh Thapa (ST) is affiliated with Jeevee Health Pvt. Ltd. However, Jeevee Pvt. Ltd. had no involvement in any form in this research project. The author has worked on this project in his capacity as an independent scholar. The author alone is responsible for the views expressed in this article, and they do not necessarily represent the views, decisions or policies of the institution with which they are affiliated.

5.1. Please provide an amended Funding Statement declaring this commercial affiliation, as well as a statement regarding the Role of Funders in your study. If the funding organization did not play a role in the study design, data collection and analysis, decision to publish, or preparation of the manuscript and only provided financial support in the form of authors' salaries and/or research materials, please review your statements relating to the author contributions, and ensure you have specifically and accurately indicated the role(s) that these authors had in your study. You can update author roles in the Author Contributions section of the online submission form.

Please also include the following statement within your amended Funding Statement.

"The funder provided support in the form of salaries for authors [insert relevant initials], but did not have any additional role in the study design, data collection and analysis, decision to publish, or preparation of the manuscript. The specific roles of these authors are articulated in the 'author contributions' section."

If your commercial affiliation did play a role in your study, please state and explain this role within your updated Funding Statement.

**Authors' Response:** The author(s) received no funding. Jeevee Health Pvt. Ltd provided support in the form of salary for author Santosh Thapa. There are no patents, products in development or marketed products associated with this research to declare. This does not alter our adherence to PLOS ONE policies on sharing data and materials.

5.2. Please also provide an updated Competing Interests Statement declaring this commercial affiliation along with any other relevant declarations relating to employment, consultancy, patents, products in development, or marketed products, etc.

Within your Competing Interests Statement, please confirm that this commercial affiliation does not alter your adherence to all PLOS ONE policies on sharing data and materials by including the following statement: "This does not alter our adherence to  PLOS ONE policies on sharing data and materials." (as detailed online in our guide for authors <http://journals.plos.org/plosone/s/competing-interests>) . If this adherence statement is not accurate and  there are restrictions on sharing of data and/or materials, please state these. Please note that we cannot proceed with consideration of your article until this information has been declared.

Please include both an updated Funding Statement and Competing Interests Statement in your cover letter. We will change the online submission form on your behalf.

Please know it is PLOS ONE policy for corresponding authors to declare, on behalf of all authors, all potential competing interests for the purposes of transparency. PLOS defines a competing interest as anything that interferes with, or could reasonably be perceived as interfering with, the full and objective presentation, peer review, editorial decision-making, or publication of research or non-research articles submitted to one of the journals. Competing interests can be financial or non-financial, professional, or personal. Competing interests can arise in relationship to an organization or another person. Please follow this link to our website for more details on competing interests: <http://journals.plos.org/plosone/s/competing-interests>

**Authors' Response:** Funding Statement and Competing Interests Statement in our cover letter and these statements are now revised.

6. We note that you have indicated that data from this study are available upon request. PLOS only allows data to be available upon request if there are legal or ethical restrictions on sharing data publicly. For information on unacceptable data access restrictions, please see <http://journals.plos.org/plosone/s/data-availability#loc-unacceptable-data-access-restrictions>.

In your revised cover letter, please address the following prompts:

a) If there are ethical or legal restrictions on sharing a de-identified data set, please explain them in detail (e.g., data contain potentially identifying or sensitive patient information) and who has imposed them (e.g., an ethics committee). Please also provide contact information for a data access committee, ethics committee, or other institutional body to which data requests may be sent.

b) If there are no restrictions, please upload the minimal anonymized data set necessary to replicate your study findings as either Supporting Information files or to a stable, public repository and provide us with the relevant URLs, DOIs, or accession numbers. Please see <http://www.bmj.com/content/340/bmj.c181.long> for guidelines on how to de-identify and prepare clinical data for publication. For a list of acceptable repositories, please see <http://journals.plos.org/plosone/s/data-availability#loc-recommended-repositories>.

We will update your Data Availability statement on your behalf to reflect the information you provide.

**Authors' Response:** We have now uploaded the data.

7. Please include captions for your Supporting Information files at the end of your manuscript, and update any in-text citations to match accordingly. Please see our Supporting Information guidelines for more information: <http://journals.plos.org/plosone/s/supporting-information>.

**Authors' Response:** We have also included captions for supporting information files at the end of the manuscript.

[Note: HTML markup is below. Please do not edit.]

**Reviewers' comments:
Reviewer's Responses to Questions**

**Comments to the Author**
1. Is the manuscript technically sound, and do the data support the conclusions?
The manuscript must describe a technically sound piece of scientific research with data that supports the conclusions. Experiments must have been conducted rigorously, with appropriate controls, replication, and sample sizes. The conclusions must be drawn appropriately based on the data presented.

Reviewer #1: Partly

Reviewer #2: Yes

**Authors' Response:** In this revised version, we have carefully re-read the methods, results and conclusion and making sure that the manuscript is technically sound and data support the conclusions.

2. Has the statistical analysis been performed appropriately and rigorously?

Reviewer #1: No

Reviewer #2: Yes

**Authors' Response:** We have revised the manuscript taking into account the reviewers' comment.

3. Have the authors made all data underlying the findings in their manuscript fully available?
The [PLOS Data policy](http://www.plosone.org/static/policies.action#sharing) requires authors to make all data underlying the findings described in their manuscript fully available without restriction, with rare exception (please refer to the Data Availability Statement in the manuscript PDF file). The data should be provided as part of the manuscript or its supporting information, or deposited to a public repository. For example, in addition to summary statistics, the data points behind means, medians and variance measures should be available. If there are restrictions on publicly sharing data—e.g. participant privacy or use of data from a third party—those must be specified.

 Reviewer #1: No

Reviewer #2: Yes

**Authors' Response:** We have now submitted the dataset underlying the findings of the study.

 4. Is the manuscript presented in an intelligible fashion and written in standard English?
PLOS ONE does not copyedit accepted manuscripts, so the language in submitted articles must be clear, correct, and unambiguous. Any typographical or grammatical errors should be corrected at revision, so please note any specific errors here.

Reviewer #1: No

Reviewer #2: No

**Authors' Response:** We have revised the manuscript taking into account of the reviewers' comment. We have taken careful consideration of the language, flow, clarity and organization of the information and its presentation in the article to improve the standard of English in the article.

5. Review Comments to the Author
Please use the space provided to explain your answers to the questions above. You may also include additional comments for the author, including concerns about dual publication, research ethics, or publication ethics. (Please upload your review as an attachment if it exceeds 20,000 characters)

Reviewer #1: In the scarcity of the instrument of focus, the authors' aim to provide the need of validated and reliable questionnaire to measure patient's satisfaction towards pharmacy services is noble and this present study could be the answer.
However, the authors do not present the study satisfactorily and there are much room for improvement.

**Authors' Response:** We have revised the manuscript according to the suggestions by you. We believe that this manuscript is improved than prior version. The justification for the study and the scope of the study findings has been elaborated concisely in the Introduction section of the manuscript.

Firstly, it seems that the manuscript was not adequately proofread before submission. The presence of basic grammatical errors in many parts of the article can be the evidence. For example, some sentences miss the full stop (for example, lines 154 and 242) and even a "sentence" is not a sentence, but just a phrase (for example, lines 146-147).

**Authors' Response:** The manuscript is now adequately proofread prior to the submission. Some grammatical errors you have mentioned plus other that we identified later have now been addressed and careful consideration has been given to the coherency and readability of the manuscript.

The subheadings are not written consistently as well as some journal or publisher name(s) in the Reference section.

**Authors' Response:** Subheadings are now written consistently. Also, journal or publishers name(s) in the Reference Section are now written consistently.

The use of however and although in a sentence could be possible, but the authors seem to not use them properly and potentially create confusion for the readers (lines 96-97).

**Authors' Response**: We have revised the lines 96-97. **[Addressed in Line 113-117 Page 6]**

Two sentences in the Introduction section are sequentially repeated in the Methods section, i.e. lines 90-93 vs 159-163.

**Authors' Response:** Regarding the sentence which was repeated in the Introduction Section and Methods section, is now removed from the Introduction Section. **[Addressed in Page No. 11 Line No.225-229]**

The reference number of ethical clearence presented in line 247 is slightly different from line 421.

**Authors' Response:** Thank you for pointing out typo error in reference number of ethical clearance in Line 247. We have now corrected in the revised version **[Addressed in Page No. 37 Line No. 674]**

Secondly, it seems that the authors want to provide a validated and reliable translated questionnaire that could be useful for all pharmacy settings, i.e. hospitals, clinics, and community pharmacies, but the sample was only taken from community pharmacies without sufficient details about the number of pharmacies, sampling method, and typical services provided by the pharmacies.

**Authors' Response:** Yes, we have taken sample only from community pharmacies. Now, we have revised the manuscript. We have excluded some of the information provided about hospital pharmacies/clinical pharmacies and have mentioned this is validated for community pharmacy setting.

The study may also be prone to recall bias as the defined recall period is 3 months.

**Authors' Response:** Thank you for raising this issue. Due to the selection criteria for the patients, it was inevitable to avoid recall bias in the study. Therefore, we have included this as one of the limitations of our study.

Providing more details on the data collection and giving more focus on community pharmacy settings (i.e specify the pharmacy setting in the title) may improve the quality of the manuscript.

**Authors' Response:** We have provided more details on data collection and gave more focus on community pharmacy settings throughout the manuscript (especially in introduction and the discussion part). We believe that addressing this comments have improved the quality of the manuscript than prior version. We have also added community pharmacy setting in the title, which now reads as *" Translation, cross-cultural adaptation and validation of Patient Satisfaction with Pharmacist Services Questionnaire (PSPSQ 2.0) into the Nepalese version in a community settings"*

Thirdly, in the Results section, there are many technical details to read the tables that usually are not supposed to be written in the paragraph. They should be put as a table caption.
**Authors' Response:** Some technical details have been removed from 'Total variance explained' **[Addressed in Line 457-473]** as these are present in Table 2 (previously table 4).

Fourthly, the authors tend to repetitively report the KMO value and the adequate number of sample, especially in the Results and Disccusions sections while it may not be necessary.
**Authors' Response:** Some repetitions of KMO values have been removed from the Results and Discussion sections. [Addressed in Line 358, 428, 436, 472, 567-568]

Fifthly, in the construct validity analysis, the authors retained six factors in the final model, but the internal consistency reliability was calculated based on the three-factor solution like the original construct. No name was given for each of six factors retained in the model.

**Authors' Response:** Each factor has its details in the Supplementary files.

No further discussion was given on the discrepancy of the number of domains found in the Nepalese version vs the original version.

**Authors' Response:** The discrepancy of the number of domains found in the Nepalese version vs the original version is now discussed in the discussion section. **[Addressed in Line 585-591]**

The authors also miss to report one important table, which is the distribution of each question to each factor with its corresponding factor loading.

**Authors' Response:** Distribution of each question to each factor with its corresponding factor loading was shown in Table 2 (previously table 4) and Figure 1 in the scree plot.

The questions should be displayed in its original language and the target language, while the authors only provide the Nepalese version in the Supplementary file.

**Authors' Response:** We have also uploaded both the English and Nepalese version as a supplementary file.

Lastly, the authors also used visual inspection of the scree plot as it complemented the Kaiser's criterion of 1 (K-1) to decide the number of factors retained. While they decided to use a six-factor solution, the inspection of scree plot shows that the kink of the plot is on the third eigenvalue. This result should suggest only two or three factors should be retained. Any alternative analysis, such as the parallel analysis, is recommended to support the decision for retaining six factors.

**Authors' Response:** Yes, of course, sharp kink (i.e., point of inflexion) was on the third factor but there was still steep kink. Therefore, we subjectively stated that the point of inflexion was on the sixth eigenvalue. We relied on the 'Total Variance Explained' in Table 4 to support this as we did not do parallel analysis.

Reviewer #2: Title: Please revise; it can be shortened (remove abbreviations for instance).

**Authors' Response:** The full form of the words from whole manuscript has been replaced with appropriate abbreviations (after expanding the abbreviated form at the first use in the manuscript. The revised title of the manuscript is now *"Translation, cross-cultural adaptation and validation of Patient Satisfaction with Pharmacist Services Questionnaire (PSPSQ 2.0) into the Nepalese version in community settings."* **[Addressed in Line 2-3, Page 1]**

Please be consistent; Napalese version vs Napalese language.

**Authors' Response:** To make a consistent throughout the manuscript, we have used the “***Nepalese”.***

Some minor english editing of the manuscript is required.
**Authors' Response:** We have now done English editing of the manuscript.

The number of tables could be reduced.

**Authors' Response:** We have now kept table 2 as a supplementary table. Some tables are removed and merged it into one. **Tables are now limited to three only.**

Introduction:
Patient vs consumer satisfaction; patients needs in many ways to rely on the service; while consumers can easily disregard the service. Is this an issue when it comes to needs and whether or not to measure satisfaction?

**Authors' Response:** The reviewer raises an important point and we are thankful for this comment.We have addressed this important on patient vs consumer satisfaction in the **DISCUSSION SECTION.** We have added following paragraph in **Line No 534-545, Page 31**

*"Patient satisfaction may not relate to consumer satisfaction, as we see it in the retail sector. In Nepal, community pharmacy is also known as a retail pharmacy. There may be two possibilities. The first possibility is that an unsatisfied consumer may not buy a product or service; it may be detrimental to business but not too patient health. The second possibility maybe retail sectors other than community pharmacy do different things to satisfy all consumers' needs; however, the same may not be the case for healthcare or community pharmacies. For community pharmacies an unsatisfied patient means he or she might not have enough information, may not like the pharmacy environment as there is no provision for privacy, in that case, the patient may not be able to express his concerns or his healthcare needs, and it may affect his health outcome. Contrary to that, a pharmacist cannot dispense whatever a patient request over the counter to make him/her happy. Patient satisfaction is a unique issue when it comes to community pharmacy services; it is an important issue. However, it needs to be defined and measured."*

Cultural differences between English and Nepalese what would be the most prominent ones?

**Authors' Response:** The cultural difference between English and Nepalese is now added in the **Introduction Section [Line No. 166-174].** The added portion is mentioned below.

*"Nepalese language is also the mother tongue of Bhutanese refugees living in Nepal. Nepal is a homeland to the people with unique culture, language, health literacy, socio-economic profile and health-seeking behaviour; and health practice in Nepal is very different from the English-speaking countries. For example, patients asking pharmacists for specific medicines may be a common thing in developed countries, as the population health literacy in those countries is higher. Contrary to that, pharmacists in developing countries like Nepal often receive requests from the patients for a medicine with a particular colour, brand and dosage form due to inadequate health literacy. Health literacy in the Nepalese people is one of the less explored areas."*

Aim; I don't think as it is currently written encompass cultural adaption.

## **Authors' Response:** Thank you for pointing out the error. After the translation we have cultural adaptation with help of expert committee. We have addressed this comment in subheading. **Step 1 – Translation procedures and cultural adaptation**

Methods:
Consistent and clearly stated.
But, from the introduction I get the impression that this tool can be used in all pharmacies; but you only collect data from community pharmacies: Perhaps it is wise to exclude some of the information provided about hospital pharmacies/clinical pharmacies; or at least this needs to be discussed thoroughly.
**Authors' Response:** Yes, we have collected the data from community pharmacies only. We have now removed the information provided about hospital pharmacies and clinical pharmacies.

The sample size seems appropriate; and the statistics is well described.
**Authors' Response:** Thank you for the encouraging comment.

Results:
The correlation matrix could be presented as a supplement in its full version.
Several of the correlation is way below 0.3, is this problematic, and should it be discussed?

**Authors' Response:** We have now kept Table 2 as the supplementary file and correlation <0.3 were briefly discussed under heading R matrix (co-relation matrix). **[Addressed in Page No. 21 Line 414-422]**

Table 3, unessecary; information could be given in text.

**Authors' Response:** We have now removed Table 3 according to your suggestions and information of Table 3 are now limited in text only.

Table 4. I suggest that you instead provide the percentages of explained variance from the different loadings.

**Authors' Response:** Percentages of explained variance from the different loadings were mentioned in Table 2 (previously table 4). **[Addressed in** **Line 457-473]**

It could also be an idea to present the scree plot before table 4; this will provide the reader with information about how many different factors one should include.

**Authors' Response:** Figure 1 is now kept before Table 4. **[Page 25]**

Discussion:
All data are collected by one pharmacist in community pharmacies: please discuss.

**Authors' Response:** Yes, the data collection was carried by one investigator in a one community pharmacy.

The cross cultural adaption; is the culture among Nepalese speaking people in India, Buthan and Myanmar similar?

**Authors' Response:** The reviewer raises an important point. As suggested, we have now added the following line. **[Addressed in Line 514-527, Page 30-31]**

*The practising pharmacists working in community settings of Nepal or specific geographical regions of other countries (e.g. part of India, Bhutan, and Myanmar) where Nepalese is spoken predominantly can be significantly benefitted by this translated, culturally adapted and validated reliable questionnaire. There are people with Nepalese ethnicity in India, Bhutan and Myanmar. They belong to the broader Nepalese community but citizens of a different country. While there might be some similarity in terms of culture, language, festival etc. when it comes to healthcare seeking practice or healthcare belief the culture or influence of culture might be different given the difference in context and access to resources and their standards of living. However, further studies are needed to comment on the overall culture and its effect on healthcare-seeking practice among Nepalese communities living Bhutan and Myanmar. Due to high mobility of people between Nepal and India, the Nepalese community living in a particular region of Northern, Eastern and North-Eastern India might have different cultural experiences when compared to the Nepalese community of Bhutan and Myanmar. Again, more studies are needed on their cultural experiences and the influence of their culture on healthcare-seeking practice or healthcare belief system.*

The number of participants in the study is good. 300 is acceptable. Please do not repeat it.

**Authors' Response:** Thank you for the comment. We have now removed this repetition from the manuscript. **[Line No. 450, 480,567, 596]**

Why is your sample not represenative; could you please elaborate on these characteristics. Please provide information about how many of the respondents that couldn't read or write. On p. 211 you state that patients filled out the questionnaire by themselves: pleas adjust wording.

**Authors' Response:** We have elaborated the sample characteristics. Line 211 is now reworded **[Addressed in Page No. 16 Line No. 333-336]** and additional information is given in the demography section of the results **(Table 1)**.
Reference list:
Please adjust/provide adequate informartion; e.g., ref no. 33.

**Authors' Response:** We have now revised this reference number 33 along with all the references in the reference list.

 6. PLOS authors have the option to publish the peer review history of their article ([what does this mean?](https://journals.plos.org/plosone/s/editorial-and-peer-review-process#loc-peer-review-history)). If published, this will include your full peer review and any attached files.
If you choose "no", your identity will remain anonymous but your review may still be made public.
**Do you want your identity to be public for this peer review?** For information about this choice, including consent withdrawal, please see our [Privacy Policy](https://www.plos.org/privacy-policy).

 Reviewer #1: No

Reviewer #2: **Yes:** Kjell H. Halvorsen

**Authors' Response:** On behalf co-authors, I would like to thank both reviewers and editor for their valuable time and encouraging comments.
